# Effects of Bojungikgi-tang on anorexic patients with atopic dermatitis: A protocol for a randomized, usual care-controlled, assessor-blinded, parallel, pilot clinical trial

**DOI:** 10.1097/MD.0000000000028965

**Published:** 2022-03-04

**Authors:** Boram Lee, Hyo-Ju Park, So Young Jung, O-Jin Kwon, Mi Mi Ko, Hyun Ah Jeong, Jeeyoun Jung

**Affiliations:** a Department of Clinical Korean Medicine, Graduate School, Kyung Hee University, Kyunghee-daero 26, Dongdaemun-gu, Seoul, Republic of Korea; b Clinical Research Coordinating Team, Korea Institute of Oriental Medicine, Yuseong-daero 1672, Yuseong-gu, Daejeon, Republic of Korea; c KM Science Research Division, Korea Institute of Oriental Medicine, Yuseong-daero 1672, Yuseong-gu, Daejeon, Republic of Korea; d Department of Korean Medicine Ophthalmology & Otolaryngology & Dermatology, Daejeon Korean Medicine Hospital of Daejeon University, Daedeok-daero 176 beon-gil 75, Seo-gu, Daejeon, Republic of Korea.

**Keywords:** anorexia, atopic dermatitis, *Bojungikgi-tan*g (*Bu Zhong Yi Qi Tang*, *Hochuekkito*), clinical trial protocol, herbal medicine, randomized controlled trial

## Abstract

**Background:**

Anorexia and atopic dermatitis (AD) are highly prevalent diseases, and the herbal medicine *Bojungikgi-tang* (BJT) has been frequently used for the treatment of both anorexia and AD. However, no study has simultaneously evaluated the effects of BJT for both anorexia and AD.

**Methods:**

A prospective, randomized, usual care-controlled, assessor-blinded. parallel, pilot clinical trial has been designed to explore the feasibility, preliminary effectiveness, and safety of BJT for the treatment of anorexic patients with AD. Forty anorexic patients with AD will be randomly assigned (1:1) to BJT or the usual care group. The BJT group will be administered BJT granules twice a day for 8 weeks and followed up for 4 weeks whereas the usual care group will not receive BJT granules. All participants in both groups will be provided with over-the-counter topical corticosteroids as a relief drug. Data will be collected at baseline and at 4, 8, and 12 weeks after randomization. The primary outcome is the score on the anorexia visual analog scale at 8 weeks post-treatment. The secondary outcomes include body weight, body fat percentage, body fat mass, skeletal muscle mass, SCORing of Atopic Dermatitis index, Validated Investigator Global Assessment scale for Atopic Dermatitis, Dermatology Life Quality Index, EuroQoL 5 Dimension 5 Level, deficiency and excess pattern identification questionnaire, total immunoglobulin E, eosinophil count, and frequency and amount of use of topical corticosteroids. Adverse events and laboratory test results will be monitored to assess safety. Fecal samples to check for gut microbiome changes and blood samples to check immune and metabolic markers will be collected before and after taking BJT.

**Discussion:**

This is the first trial that explores the preliminary effectiveness and safety of BJT for the treatment of anorexic patients with AD. The results of this pilot study will provide the basic evidence for large-scale, confirmatory, multicenter, high-quality clinical trials.

**Trial registration:**

Clinical Research Information Service, KCT0006784 (registered on November 26, 2021).

## 1 Introduction

Anorexia is a representative digestive symptom. The digestive system not only digests food and absorbs and supplies energy and nutrients to the body and skin. but also accounts for 70% of the immune system. Therefore, abnormalities of the digestive system, such as indigestion, anorexia, and the leaky gut syndrome, can cause not only digestive symptoms but also immunological disorders, such as atopic dermatitis (AD).^[1]^ A recent study showed that imbalance of the intestinal microflora, due to intestinal inflammation and dysregulation of the intestinal epithelial barrier, leads to chronic progression of AD, which is a chronic, recurrent inflammatory skin disease and a representative allergic disorder.^[2,3]^

Currently, there is no specific allopathic medical treatment for anorexia without an organic cause. Although the use of gastrointestinal motility-modulating agents, probiotics, vitamins, zinc, and iron have been recommended in clinical practice, their effectiveness has been insufficiently verified and some adverse events, such as abdominal cramps and skin rashes, have been reported.^[4]^ Furthermore, corticosteroids and calcineurin inhibitors are used to temporarily relieve itching in AD, although concerns persist about side effects, such as capillary dilatation, redness, and skin atrophy.^[5]^ Therefore, interest in herbal medicines which are derived from natural products that are relatively safe and have multi-component, multi-target, and multi-pathway characteristics is increasing, and related research is being actively conducted.^[6–8]^

Experimental studies have reported the effects of *Bojungikgi-tang* (BJT; *Bu Zhong Yi Qi Tang* in Chinese and *Hochuekkito* in Japanese) on digestive aspects, such as gastric protection and intestinal motility,^[9–11]^ as well as on the alleviation of dermatitis-related symptoms and reduction of blood immunoglobulin E (IgE) levels in an AD model.^[12]^ A clinical study demonstrated that BJT administration for 6 months in AD patients with anorexia, low fever, excessive sweating, and palpitations significantly decreased the use of topical steroids and immunomodulators than in the placebo group.^[13]^ However, no study has evaluated the effects of BJT on symptoms of both anorexia and AD symptoms in AD patients with anorexia.

Therefore, the aim of this study is to explore the feasibility, preliminary effectiveness, and safety of BJT for the treatment of anorexic patients with AD.

## 2 Methods/design

### 2.1 Trial design and recruitment

**Figure 1:**
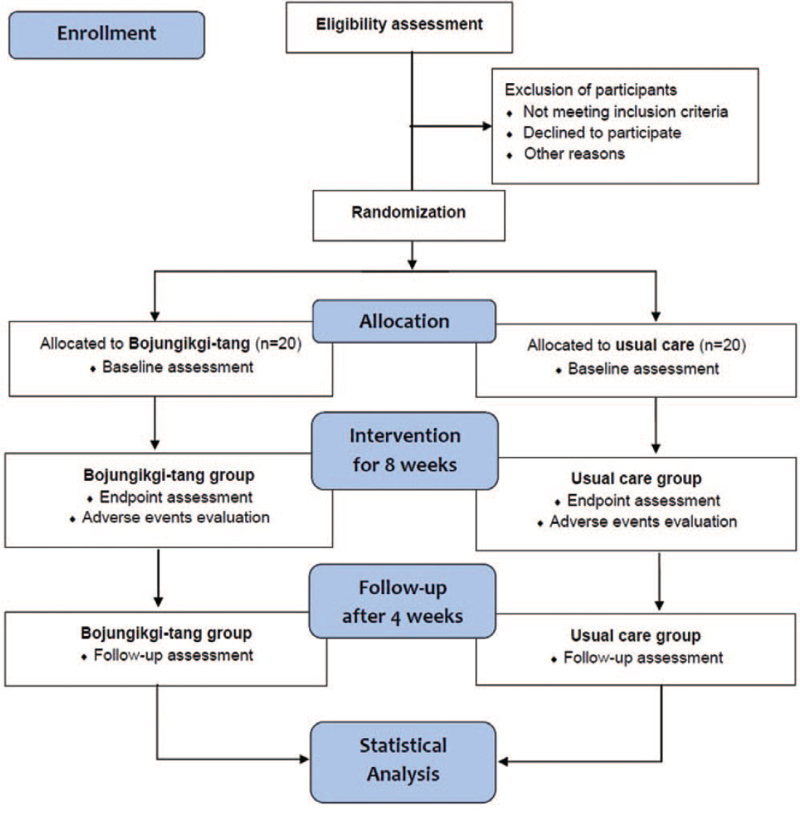
CONSORT 2010 Flow Diagram.

A prospective, randomized, usual care-controlled, assessor-blinded, parallel, pilot clinical trial will be performed at the Daejeon Korean Medicine Hospital of Daejeon University (Daejeon, Republic of Korea). Eligible participants will be recruited by inviting interest in study participation through online clinical trial posters (hospital webpages, clinical trial-related websites, etc.) and offline advertisements (public transportation, bulletin boards, etc.) from December 2021 to December 2023, during the estimated recruitment period. The clinical trial period consists of a 2-week screening period, followed by a 8-week medication program and a 4-week follow-up period for each participant (Fig. 1). Investigators will inform each eligible participant about the objectives, study procedures, and potential benefits and risks, and informed consent will be obtained from all participants prior to their enrolment in the study. Participants will be informed that they can withdraw midway at any point in the study without any consequences. In addition, the personal information of participants will be de-identified and securely stored for access by only authorized research personnel. The researchers will ensure that the trial is conducted in accordance with the tenets underlying the Declaration of Helsinki and the Good Clinical Practice Guidelines.

### 2.2 Eligibility criteria

#### 2.2.1 Inclusion criteria

1. adults aged 19 to 65 years
2. intensity of anorexia ≥40 points on a 0 to 100 mm visual analog scale (VAS) measurement
3. diagnosis of AD based on the Hanifin and Rajka diagnostic criteria^[14]^
4. mild to moderate AD according to the SCORing of Atopic Dermatitis (SCORAD) Index (score 15–50)
5. voluntary provision of written informed consent approved by the institutional review board (IRB)

#### 2.2.2 Exclusion criteria

1. presence of an organic cause of anorexia or alarming symptoms, such as rapid weight loss of 10% or more within the last 6 months
2. use of an appetite stimulant or health functional food (other than lactobacilli) or receiving Korean medical treatment for improving anorexia within the last 2 weeks
3. presence of severe skin diseases other than AD
4. use of oral corticosteroid, oral antibiotics, or systemic immunosuppressants, or receiving systemic photochemotherapy within the last 4 weeks; use of antihistamines, topical corticosteroids, topical immunomodulators, or topical antibiotics within the last 1 week; and ingestion of health functional food (excluding lactobacilli) or receiving Korean medical treatment for improving AD within the last 2 weeks
5. commencement of lactobacilli ingestion within the last 2 weeks
6. presence of serious medical conditions that preclude participation in this clinical trial

6-1. diagnosed with a mental illness, such as anorexia nervosa, depression, or anxiety disorder
6-2. uncontrolled hypertension (systolic blood pressure ≥180 mm Hg; diastolic blood pressure ≥110 mm Hg at screening)
6-3. uncontrolled diabetes (glycated hemoglobin (HbA1c) ≥9% or fasting blood glucose ≥160 mg/dL at screening)
6-4. severe liver or renal disease (aspartate aminotransferase or alanine aminotransferase levels ≥3 times the upper limit of normal or creatinine levels ≥2 times the upper limit of normal)
6-5. past or present malignancy
6-6. serious uncontrolled systemic diseases, including cardiovascular, respiratory, digestive, genitourinary, neurological/psychiatric, and hematological diseases, or serious infectious diseases

7. a history of serious alcohol abuse or drug abuse
8. presence of genetic conditions, such as galactose intolerance, Lapp lactase deficiency, or glucose-galactose malabsorption
9. known hypersensitivity to investigational products
10. use of other investigational products within the last 4 weeks
11. women who are pregnant or lactating, or women who do not agree to use effective methods of contraception during the clinical trial
12. participants who are judged by investigators to be inappropriate for participating in this study

### 2.3 Randomization and allocation concealment

A medical statistician who is uninvolved in the intervention and evaluation in this study will generate a random number table using the statistical program SAS Version 9.4 (SAS institute. Inc, Cary, NC). Block randomization without stratification will be conducted; Block size 4 will be selected and a sequence that can be assigned to each block size will be randomly chosen. The allocation ratio for each group is 1:1. The unique code for each participant according to the random number table will be placed in an opaque sealed envelope, and the labeling will remain the same for each group. Each participant who meets all eligibility criteria is assigned to a test or control group by the investigator according to a pregenerated random number table, and the investigators will open each envelope in front of the enrolled participant at the baseline visit (Week 0). The opened envelope will be marked with the date of opening and the investigator's signature and kept separately.

### 2.4 Blinding

As it is impossible to blind the participants because of the trial design (BJT vs usual care), this study is designed to blind the outcome assessor in order to minimize the bias related to the clinical trial. The evaluation of the effects on participants will be conducted by an investigator who does not prescribe investigational products and does not have access to the source documents, such as electronic medical records and case report forms. The blinded outcome assessor asks questions and evaluates participants only to assess the treatment effectiveness.

### 2.5 Interventions

Participants who fulfil the eligibility criteria at the screening visit will visit the hospital within 2 weeks and be randomized by investigators to either the BJT or the usual care group. Participants in the BJT group will consume 3.75-g BJT granules twice a day before or between meals for 8 weeks in accordance with a dosage regimen that has been established based on the regulatory approval from the Korean Ministry of Food and Drug Safety. We will use Kracie BJT Extract Fine Granules manufactured by Kracie Pharma Korea Co., Ltd (Seoul, Republic of Korea) in accordance with good manufacturing practice standards. The BJT granules are light yellowish-brown to brown fine granules comprising the extracts of the following 10 medicinal plants: *Ginseng Radix* 4.0 g, *Atractylodis Rhizoma Alba* 4.0 g, *Astragali Radix* 4.0 g, *Angelicae Gigantis Radix* 3.0 g, *Zizyphi Fructus* 2.0 g, *Bupleuri Radix* 2.0 g, *Citri Unshius Pericarpium* 2.0 g, *Glycyrrhizae Radix et Rhizoma* 1.5 g, *Cimicifugae Rhizoma* 1.0 g, and *Zingiberis Rhizoma Recens* 0.5 g (per 1 day). A pharmacist is responsible for ensuring the storage, dispensation, and quality control of the investigational products.

The usual care group will receive conventional care without BJT during the trial period. Compensation (for 2 weeks of BJT), if desired by the participants, is provided after the clinical trial is completed. However, during the examination by a Korean medical doctor, if it is ascertained that the compensation would prove damaging to the participants’ health, then compensation may not be made.

All participants in both groups will be provided an over-the-counter topical corticosteroid (Lidomex Cream 0.15%; Prednisolone Valeroacetate 1.5 mg/g) as a relief drug, and will be educated to apply an appropriate amount (fingertip unit: a quantity that fits on 1 index finger for a lesion that approximately matches the area of 2 adult palms). When applying the external remedy, participants should write the date, time of use, site of use, and amount used in the diary as well as return the remaining cream at each visit. The investigator will measure the remaining amount to 2 decimal places through a precision balance to record the amount of topical corticosteroid use in both groups during the study period.

Concomitant administration of drugs that are judged by the investigator to have no effects on the interpretation of the results of the trial will be permitted. The product name, the purpose of administration, total daily dose, route of administration, and duration of administration of the drugs administered concurrently during the study period must be recorded in the case report forms. During the study period, if it is necessary to administer the following prohibited drugs due to the circumstances of the participants, the participants will be dropped from the trial: appetite stimulants, antihistamines, oral antibiotics, corticosteroids, immunosuppressants, systemic photochemotherapy, and other drugs that are judged by investigators to affect the interpretation of the results of this clinical trial. In addition, the administration of health functional food or lactobacilli and Korean medical treatment for the treatment of anorexia or AD will be prohibited. However, oral antibiotics are allowed if it is judged that the short-term antibiotic therapy, for the treatment and management of diseases other than skin diseases, will not affect the interpretation of the trial results. In addition, if the participant has been taking lactobacilli-containing formulations continuously for 2 weeks before the screening visit, the lactobacilli use is allowed as long as the dose or type is not changed during the study period.

### 2.6 Outcome measures

#### 2.6.1 Primary outcomes

The primary outcome measure is the self-reported score on the horizontal 0 to 100 cm anorexia VAS after 8 weeks of treatment (a primary endpoint) that participants themselves use to indicate the intensity of subjective anorexia symptoms over the past 2 weeks. A VAS of 0 means no anorexia-related discomfort, and a score of 100 means the highest discomfort imaginable. The VAS score will be assessed at all visits except at the baseline visit.

#### 2.6.2 Secondary outcomes

The secondary outcomes include body weight, body fat percentage, body fat mass, skeletal muscle mass, SCORAD Index, Validated Investigator Global Assessment Scale for Atopic Dermatitis, Dermatology Life Quality Index (DLQI), EuroQoL 5 Dimension 5 Level (EQ-5D-5L), Deficiency and Excess Pattern Identification Questionnaire (DEPIQ), total IgE, eosinophil count, and frequency of use and dosage of remedies.

Body weight, body fat percentage, body fat mass, and skeletal muscle mass will be measured using body composition-measuring equipment at every visit except the screening visit. The SCORAD Index, developed by the European AD Task Force Team, is a reliable evaluation index for assessing the severity of AD.^[15]^ The evaluation method is divided into 3 categories: the extent of the lesion (A), the intensity of the lesion (B), and the subjective symptoms (C), and the index is calculated according to the score summation method (SCORAD Index = A/5 + 3.5B + C). The extent of the lesion is calculated from 0% to 100% using the rule of nine, and the intensity is graded from 0 to 3 (0 = absence, 1 = mild, 2 = moderate, 3 = severe, respectively) for erythema, edema/papulation, oozing/crust, excoriation, lichenification, and dryness. Subjective symptoms will be elicited to ascertain the degree of pruritus and sleep loss for the past 3 days on a 0 to 10 cm VAS. The score range of the SCORAD Index is 0 to 103, with a higher score indicating more severe AD symptoms. The SCORAD Index will be assessed at every visit except the baseline visit. Each of the SCORAD total score, objective score (the extent and intensity of the lesion), and subjective score (pruritus and sleep loss) components will be adopted as evaluation variables. The Investigator Global Assessment Scale is a measure to evaluate the overall disease severity for clinical symptoms, and the reliability and validity of the Validated Investigator Global Assessment scale for Atopic Dermatitis in the evaluation of AD has been confirmed.^[16]^ The investigator will evaluate the overall improvement of the participant's AD symptoms on a 5-point scale (0–4 points) at all visits except the screening visit. The DLQI is an evaluation tool that comprises 10 items to measure the quality of life for dermatological diseases, and its validity has been previously confirmed.^[17,18]^ Each item of the DLQI is scored on a scale of 0 to 3, and a higher score indicates a lower quality of life. The DLQI will be assessed at every visit except the screening visit. The EQ-5D-5L is a self-reported questionnaire for assessing the quality of life of the general population and of patients with various diseases; the EQ-5D-5L assesses mobility, self-care, usual activities, pain/discomfort, and anxiety/depression on a 5-point scale.^[19]^ The health status will be evaluated using the EuroQoL Visual Analog Scale, scored from 0 (the worst imaginable health state) to 100 (the best imaginable health state), using vertical lines at every visit except the screening visit. The DEPIQ is a self-reported questionnaire that consists of 20 questions each related to the deficiency and excess patterns,^[20,21]^ and the respective scores will be adopted as outcome variables. The DEPIQ score will be evaluated at the baseline visit (Week 0) and at Visit 4 (Week 8). In addition, to evaluate hematological biomarkers related to AD, the total IgE level and eosinophil counts will be evaluated at the screening visit and at Visit 4 (Week 8). Furthermore, we evaluate the frequency of use and amount of topical corticosteroids remedies distributed by reviewing the diary maintained by participants on the use of remedies at Visit 3 (Week 4), Visit 4 (Week 8), and Visit 5 (Week 12) as shown in Table 1.

**Table 1:** Schedule of enrolment, interventions, and assessments.

	Study period				
	Screening	Postallocation			Follow-up
TIME POINT	∼ 2 weeks	Week 0	Week 4(± 4 days)	Week 8(± 4 days)	Week 12(± 4 days)
ENROLMENT					
Informed consent	•				
Eligibility screening	•				
Demographics and medical history taking	•				
Randomized allocation		•			
INTERVENTIONS					
BJT administration^†^		←–- - - - - - - - - - - - - - - - - - - - - - - →			
Distribution of topical corticosteroids (remedy) and usage diary		•	•	•	
ASSESSMENTS					
Vital signs	•	•	•	•	•
Physical examination	•				
Hanifin and Rajka Criteria	•				
Atopic dermatitis pattern identification questionnaire		•			
Laboratory test^∗^	•			•	
Fecal test (gut microbiome)		•		•	
Anorexia VAS	•		•	•	•
Body weight, body fat percentage, body fat mass, skeletal muscle mass		•	•	•	•
SCORAD	•		•	•	•
vIGA-AD		•	•	•	•
DLQI		•	•	•	•
EQ-5D-5L		•	•	•	•
DEPIQ		•		•	
3-day food record		•		•	
Adverse events		•	•	•	•
Compliance of investigational product^†^			•	•	

BJT = Bojungikgi-tang, DEPIQ = deficiency and excess pattern identification questionnaire, DLQI = Dermatology Life Quality Index, EQ-5D-5L = EuroQoL 5 Dimension 5 Level, SCORAD = SCORing of Atopic Dermatitis, VAS = visual analog scale, vIGA-AD = Validated Investigator Global Assessment scale for Atopic Dermatitis.

∗ hematological test, blood glucose parameters, liver and renal function test, electrolyte, total immunoglobulin E, and blood immunity and metabolic indicators; human chorionic gonadotropin urine test only for women in their childbearing years at the screening visit.

† Only in the BJT group.

#### 2.6.3 Safety outcomes

Laboratory tests of liver (aspartate aminotransferase and alanine aminotransferase) and renal function (blood urea nitrogen and creatinine) profiles will be conducted for all participants at the screening visit and at Visit 4 (Week 8) to assess the safety of the BJT. In addition, at each visit, reports of adverse reactions are collected through the participant's symptom report as well as the investigator's observation during clinical examination. Descriptive explanations, including severity, causality, and predictability and the frequency, of all adverse events that have occurred should be recorded and will be analyzed. Investigators will educate the participants on all possible adverse reactions that may occur after BJT administration and on the reporting of all symptoms that occur during the study period. In addition, all participants with adverse reactions will receive appropriate medical treatment until the disappearance of the symptoms.

#### 2.6.4 Study feasibility outcomes

The recruitment rate (a percentage of the number of enrolled participants relative to the total number of screened participants), adherence rate (a percentage of the number of participants completing 70% or more of the investigational product administration schedule relative to the total number of enrolled participants in BJT group), and completion rate (a percentage of the number of participants who complete the trial without dropping out relative to the total number of enrolled participants) will be calculated to determine whether a confirmatory, full-scale, randomized controlled trial is feasible.

#### 2.6.5 Exploratory outcomes

Multiplex-based Th1/Th2 immune marker analysis will be performed on blood samples obtained before (screening visit) and after the administration of the investigational product (Week 8). In addition, 3000 to 5000 detectable metabolites are profiled through nuclear magnetic resonance/mass spectrometry-based serum metabolomics analysis. Fecal samples of participants that were obtained before (Week 0) and after the administration of the investigational product (Week 8) will be examined to identify changes in the gut microbiome through next-generation sequencing. As the participants’ food intake is predicted to act as a factor that can affect the intestinal microbiome, participants will submit a food record diary of the type and amount of food intake for 3 days prior to the baseline visit (Week 0) and until Visit 4 (Week 8). Nutrient analysis will be performed on food contents using CAN Pro 5.0 (The Korean Nutrition Society, Seoul, Republic of Korea).

### 2.7 Sample size

This is the first preliminary exploratory study to assess the improvement of anorexia and AD symptoms of the BJT group compared to the usual care group in participants with coexisting anorexia and AD. According to a study that recommended a minimum sample size for the preliminary study,^[22]^ the minimum effect size was assumed to be 0.30, and a dropout rate of 20% was taken into consideration; a total of 40 participants, 20 in each group, was set as the target sample size.

### 2.8 Statistical analysis

All statistical analyses will be conducted using SAS Version 9.4 (SAS institute. Inc., Cary, NC). For the effectiveness analysis, a full analysis set analysis including all data obtained from participants who undergo an outcome measurement at least once after randomization will be conducted in accordance with the intent-to-treat principle. If necessary, a per protocol set analysis will be performed for participants who have completed the entire process as specified in the clinical trial protocol and have no significant violations affecting the trial results. Participants who have consumed the prohibited drugs for the treatment and management of diseases other than skin diseases at the discretion of the investigator during the study period will be excluded from the per protocol set analysis and then analyzed. The safety analysis will include all data obtained from participants who ingest the investigational product at least once.

For categorical data, the frequency and percentage are presented, whereas the mean and 95% confidence intervals are presented for continuous data. Depending on the normality, the presence of any statistically significant intergroup differences will be tested using the Chi-Squared or Fisher exact test for categorical data, and by the independent *t*-test or Wilcoxon rank sum test for continuous data. A two-sided test with a significance level of 0.05 will be performed using an analysis of covariance, with the baseline as the covariate and the treatment group as fixed factors. In addition, the analysis can be performed by setting variables in demographics or social characteristics that show statistically significant differences or by using variables that can affect anorexia and AD as covariates. The multiple imputation method will be used for processing missing data. If possible, subgroup analysis may be performed according to the sex, deficiency and excess pattern identification, remedy use, IgE levels as well as the morbidity period, pattern identification,^[23]^ and severity of AD. Intragroup changes in the outcome measures during the study period will be analyzed using the Chi-Squared or Fisher exact test for categorical data and the paired *t*-test or Wilcoxon signed rank test for continuous data. In addition, repeated measures analysis of variance will be performed to compare the difference in trend changes by visit in each group.

### 2.9 Data monitoring and management

A clinical research associate at the Korea Institute of Oriental Medicine (KIOM), which sponsored this study, will visit the institution regularly to monitor protocol compliance, recruitment rate, document, and adverse events during the trial period. The monitoring procedures and schedule will adhere to the KIOM monitoring standard operating procedure. All detected issues will be properly resolved through discussions with the investigators. Currently, no audits have been planned.

An online electronic CRF iCReaT developed by the Korea National Institute of Health (Osong, Republic of Korea) will be used for data collection and verification and will be managed by an independent data management team from KIOM. Data entry is performed only once by the investigator at the study center; however, the data quality is managed through 2 validation processes at KIOM: by each clinical research associate as well as by the data management team.

### 2.10 Ethical consideration

The trial protocol has been approved by the IRB of Daejeon Korean Medicine Hospital of Daejeon University (approval number: DJDSKH-21-DR-18) and registered at the Clinical Research Information Service (CRIS; registration number: KCT0006784). If the protocol requires modification, it will be revised and reapproved by the IRB prior to implementation and documented in the Clinical Research Information Service. Investigators (licensed Korean medical doctors) will describe the overall content and process of the trial, the potential benefits and risks, and other therapeutic options to all participants. The completed informed consent form will be obtained from each participant prior to their participation in the trial.

## 3 Discussion

This is a study protocol for a randomized, usual care-controlled, assessor-blinded, parallel, pilot clinical trial to assess the effect of BJT on patients with coexisting anorexia and AD. We will evaluate the preliminary effectiveness and safety of BJT, compared to the usual care, and evaluate the feasibility of large-scale randomized controlled trials.

Anorexia and AD are diseases with high prevalence, and there is a high demand for complementary and integrative medical treatments, including herbal medicine due to controversy over conventional treatments.^[4,5]^ In particular, it has been recently reported that the imbalance of the intestinal microflora leads to the chronic progression of AD due to intestinal inflammation and dysregulation of the intestinal epithelial barrier.^[2,3]^ This factor has generated increasing interest in therapeutics that simultaneously target gastrointestinal diseases and AD. Herbal medicine contains multiple active components that act on multiple targets and therefore has the potential to overcome the limitations of conventional medication with a single active constituent.^[24]^ Accordingly, studies of herbal medicine treatment targeting AD patients with digestive symptoms are being actively conducted.^[7,8]^

BJT is an herbal formula that has been approved by the Korean Ministry of Food and Drug Safety and is commercially marketed for the following symptoms: weak constitution, tiredness, weakness after illness, anorexia, and night sweating. In addition, according to a systematic review of the use of BJT for the treatment of AD, there were statistically significant improvements in the oozing and crust score and the total equivalent amount of topical agents when using BJT compared to placebo.^[25]^ However, no clinical trial has evaluated the effect and safety of BJT in patients with both anorexia and AD. Our study will provide scientific evidence of the effectiveness and safety of BJT for the treatment of patients with anorexia and AD through a rigorously designed clinical trial.

In this clinical trial, based on the approval by the Korean Ministry of Food and Drug Safety, we scheduled the BJT to be taken as 1 pack (3.75 g) twice a day before or between meals. In accordance with the results of a clinical trial in which BJT was administered for 6 months^[13]^ and based on consultation with clinical experts for anorexia and AD, the administration of BJT for 8 weeks was judged to have no major safety problems, and was set as the administration period for this clinical trial.

Furthermore, the SCORAD score range was set by referring to the existing herbal medicine clinical trial for patients with AD and digestive diseases.^[7,8]^ Considering that this is a pilot study to confirm the exploratory effect and feasibility of BJT administration, we referred to a study that targeted SCORAD scores of 15 or more and less than 50 points^[7]^ and another study that targeted SCORAD scores of between 25 or more and 50 or lower,^[8]^ and predefined a wide range of SCORAD score between 15 or more and up to 50 as an inclusion criterion.

The limitation of this study is that, due to the nature of the trial design, blinding of the study participants and the investigator is impossible. However, we will do our best to maintain the high quality of the trial by ensuring the blinding of the outcome assessor. In addition, the efficacy of BJT cannot be confirmed because we will use usual care rather than placebo as a control group. However, the purpose of preliminary testing is to determine the effectiveness of BJT in the real-world clinical settings wherein efficacy trials without usual care arms have been considered to have a fundamental flaw.^[26]^

To the best of our knowledge, this is the first study to evaluate the feasibility and preliminary effect of BJT on anorexic patients with AD using validated questionnaires to determine the severity of anorexia and AD as well as the health-related quality of life. In addition, we would like to examine changes in objective outcome measures, such as anthropometric values and AD-related hematological biomarkers. Furthermore, the changes in blood immunity and metabolic indicators as well as in intestinal microbiota before and after BJT administration will be explored because the effects of herbal medicines as probiotics that promote the growth of some intestinal microflora have also been reported.^[27]^ If the treatment effect and safety of BJT compared with the usual care group is demonstrated through this pilot study, the findings are expected to provide basic evidence for the development of large-scale, confirmatory, multicenter, high-quality randomized controlled trials to obtain definitive conclusions.

## Author contributions

**Conceptualization:** Boram Lee, Jeeyoun Jung.

**Data curation:** So Young Jung, O-Jin Kwon.

**Formal analysis:** Mi Mi Ko.

**Investigation:** Boram Lee, Hyo-Ju Park, Hyun Ah Jeong.

**Methodology:** Boram Lee, Jeeyoun Jung.

**Project administration:** Jeeyoun Jung.

**Supervision:** Jeeyoun Jung.

**Writing – original draft:** Boram Lee.

**Writing – review & editing:** Hyo-Ju Park, So Young Jung, O-Jin Kwon, Mi Mi Ko, Hyun Ah Jeong, Jeeyoun Jung.
